# Cuff-Less Methods for Blood Pressure Telemonitoring

**DOI:** 10.3389/fcvm.2019.00040

**Published:** 2019-04-30

**Authors:** Dylan M. Bard, Jeffrey I. Joseph, Noud van Helmond

**Affiliations:** ^1^Sidney Kimmel Medical College, Thomas Jefferson University, Philadelphia, PA, United States; ^2^Department of Anesthesiology, Sidney Kimmel Medical College, Thomas Jefferson University, Philadelphia, PA, United States; ^3^Department of Anesthesiology, Cooper Medical School of Rowan University, Cooper University Hospital, Camden, NJ, United States

**Keywords:** hypertension, telemedicine, blood pressure telemonitoring, pulse transit time, tonometry

## Abstract

Blood pressure telemonitoring (BPT) is a telemedicine strategy that uses a patient's self-measured blood pressure (BP) and transmits this information to healthcare providers, typically over the internet. BPT has been shown to improve BP control compared to usual care without remote monitoring. Traditionally, a cuff-based monitor with data communication capabilities has been used for BPT; however, cuff-based measurements are inconvenient and cause discomfort, which has prevented the widespread use of cuff-based monitors for BPT. The development of new technologies which allow for remote BP monitoring without the use of a cuff may aid in more extensive adoption of BPT. This would enhance patient autonomy while providing physicians with a more complete picture of their patient's BP profile, potentially leading to improved BP control and better long-term clinical outcomes. This mini-review article aims to: (1) describe the fundamentals of current techniques in cuff-less BP measurement; (2) present examples of commercially available cuff-less technologies for BPT; (3) outline challenges with current methodologies; and (4) describe potential future directions in cuff-less BPT development.

## Introduction

Hypertension has been well established as a leading risk factor for cardiovascular morbidity and mortality (1). The lifetime risk of developing stage two hypertension, ≥140 mmHg systolic or ≥90 mmHg diastolic based on the 2017 American Heart Association guidelines for hypertension, is nearly 90% (2). Importantly, intensive blood pressure (BP) control has been shown to significantly reduce cardiovascular morbidity and all-cause mortality associated with hypertension (3). For these reasons, frequent measurement of BP is recommended in patients with hypertension (4, 5).

BP telemonitoring (BPT) has recently emerged as a promising e-health treatment in hypertension management. BPT involves self-measurement of BP and transmission of the data to the patient's physician, who then can adjust medical therapy accordingly. BPT allows for greater patient autonomy, convenience, and perceived control over management of their chronic condition (6). Most importantly, several large meta-analyses have demonstrated that home BPT may be more efficacious at lowering both systolic and diastolic BP while normalizing BP in a larger proportion of patients vs. traditional office-based care (7–9). The effect of BPT is largely dependent on co-interventions such as medication titration by health care providers, stressing the importance of the data transmission component of BPT (10).

Traditionally, BPT is accomplished using an automated upper-arm cuff BP monitor with data transmission capabilities (11). However, these cuff-based methods have proven to be a barrier to widespread use of BPT due to discomfort associated with measurements and the rather bulky equipment that is needed (12). A more simple and user-friendly BP measurement device would enable more widespread use of BPT in hypertension management (13).

Several BP measuring devices have recently become commercially available that can measure BP without a cuff, giving patients a more user-friendly alternative to the traditional cuff (Table 1). This article aims to: (1) describe the fundamentals of current techniques in cuff-less BP measurement; (2) present examples of commercially available cuff-less technologies for BPT; (3) outline challenges with current methodologies; and (4) describe potential future directions in cuff-less BPT development.

**Table 1:** Cuff-less devices for BP monitoring.

	**BP measurement method**	**Calibration needs**	**Vital sign measurements provided**	**Duration of measurement**	**Connectivity**	**Level of validation**	**Cost**
**SMARTPHONE APPLICATIONS**							
My BP Lab	PTT	Required with validated cuff-based device before use	SBP, DBP	10 s	Users have to consent to be a part of a research study on the app when using it. Data is shared with researchers at UCSF	No published validation studies	Free to download from Google Play store
Instant Blood Pressure	Not disclosed	Not required	SBP, DBP	10 s	Data stored on smartphone local memory	Did not meet AAMI guidelines in non-ambulatory setting (17)	$4.99^*^
**WEARABLES**							
Heartisans Watch	PTT	Required with validated cuff-based device before first use	SBP, DBP, HR, HR, EKG, Pedometer	20 s	Connects with smartphone application through Bluetooth	No published validation studies	$150
BPro	Tonometry	Required with validated cuff-based device before use	SBP, DBP, HR, Pulse Waveform	Continuous measurement	Data sent to secure cloud server through bluetooth	AAMI and ESH validated in non-ambulatory setting and in ambulatory setting modest agreement with oscillometric cuff (22, 23)	$2,000
**TRICORDERS**							
Freescan	PTT	Not required	SBP, DBP, HR, EKG, Pulse waveform	10 s	Connects with smartphone through Bluetooth, data can be stored on a cloud server	AAMI validated in non-ambulatory setting	$225
Bodimetrics	PTT	Required with validated cuff-based device before use; recalibration every three months	SBP, HR, SpO_2_, EKG, Temperature, Pedometer	20 s	Connects with smartphone through Bluetooth	Failed to meet ESH guidelines in unconventional non-ambulatory validation study (25)	$299
SOMNOtouch	PTT	Required with validated cuff-based device before first use	SBP, DBP, HR, SpO_2_, EKG, Sleep Patterns (Actigraph)	Continuous measurement	Data can be transferred to a PC via a USB docking station or sent to a tablet via BlueTooth	ESH validated in non-ambulatory setting (28)	$5,200

* *Not for sale anymore. BP, blood pressure; PTT, Pulse Transit Time; SBP, systolic blood pressure; DBP, diastolic blood pressure; EKG, electrocardiogram; HR, hearty rate; SpO_2_, oxygen saturation; UCSF, University of California, San Francisco; AAMI, Association for the Advancement of Medical Instrumentation; ESH, European Society of Hypertension*.

### Cuff-Less BP Measurement Solutions

#### Smartphone Applications

A smartphone is a mobile phone that performs many of the functions of a computer, typically having a touchscreen interface, internet access, and an operating system capable of running downloaded apps. Seventy-seven percent of Americans currently own a smartphone, and smartphones offer a great potential to expand BPT use if their sensors can be manipulated to measure BP (14). The application of mobile devices in healthcare is often referred to as m-health (15).

**Figure 1:**
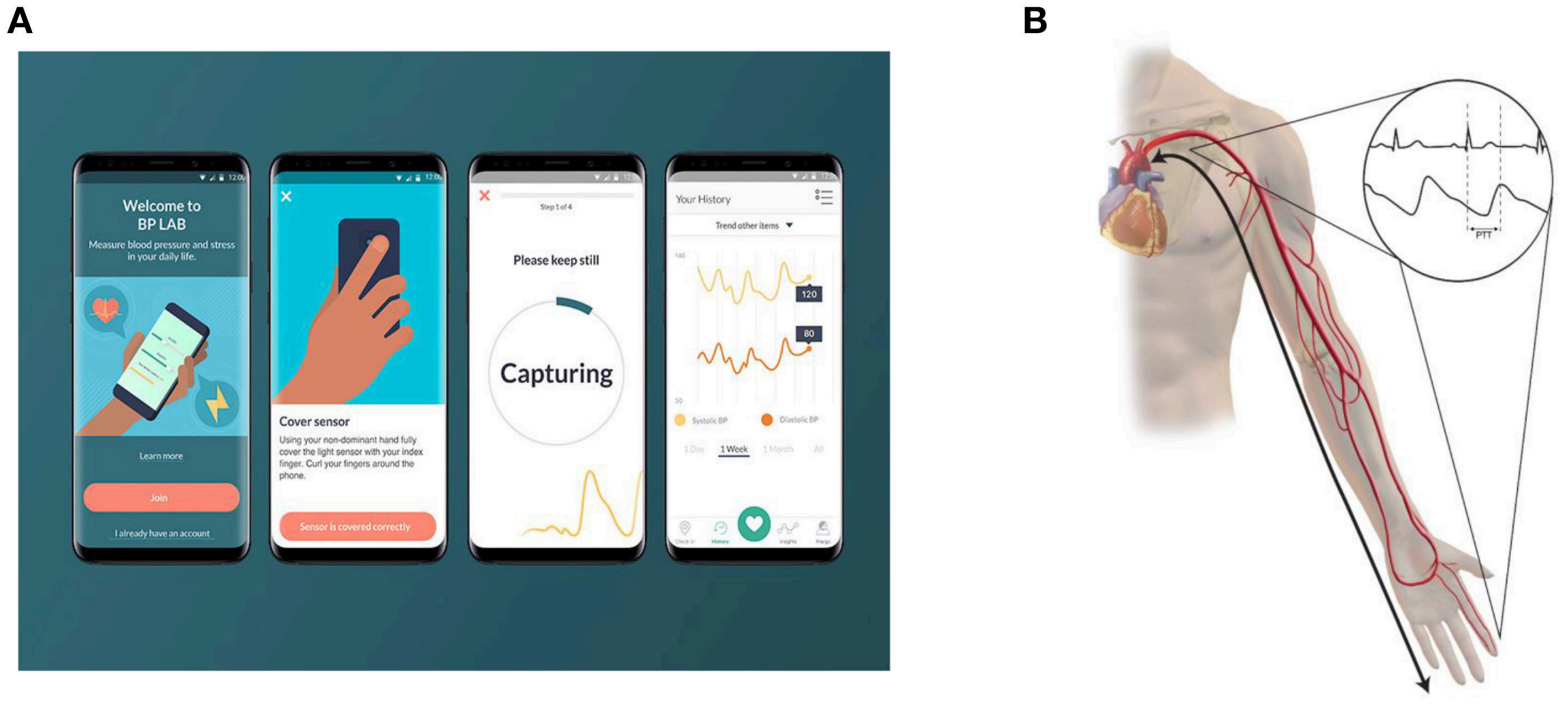
Pulse transit time (PTT) based cuff-less blood pressure measurement. **(A)** depicts the My BP Lab App that has been developed by Samsung and the University of California San Francisco. To measure blood pressure, users place their index finger on the optical sensor on the back of the phone. **(B)** depicts the underlying principle of PTT based blood pressure measurement. PTT represents the time it takes for a pulse pressure wave to travel from its origin at the heart to a distal point. Since it is difficult to non-invasively measure a pulse waveform at the heart, the onset of the QRS complex is measured as a surrogate. The arrival of a pulse pressure wave at the finger or wrist can be measured using a photoplethysmography sensor.

Smart phone applications that help users track BP values longitudinally (measured with another device) have been around for some time, but applications that actually enable the smartphone itself to measure BP are relatively novel. A 2015 survey of mobile phone applications developed for hypertension management found that seven applications claimed to measure BP; the public's interest in these applications is demonstrated by the fact that some of these applications had been downloaded almost one million times in 2015 (16). The manufacturers of these applications typically do not disclose how the measurements are performed, and the accuracy of these measurements is currently mostly unknown. A formal validation study on one particular iOS application, the Instant Blood Pressure app, revealed poor accuracy of BP measurements (17). Researchers found mean absolute differences of 12.4 mmHg for systolic BP and 10.1 mmHg for diastolic BP between the Instant Blood Pressure application and a reference device. This correlated to approximately four out of five hypertensive individuals being falsely classified as normotensive. More recently, Samsung announced a collaboration with the University of California, San Francisco, to develop the My BP Lab application that utilizes a built-in optical sensor in the Galaxy S9 mobile phone to measure BP (Figure 1A) (18). The My BP Lab application measures BP through measurements of pulse transit time (PTT). PTT refers to the time required for the arterial pressure wave to travel from the left ventricle to a distal arterial site (Figure 1B). PTT is inversely related to blood pressure and dependent on arterial compliance, arterial wall thickness, arterial radius, and blood density (19). The inter-individual differences in these parameters necessitate initial calibration of PTT-based BP measurements to a cuff-based BP monitor (19). PTT is typically calculated using electrocardiogram and photoplethysmography sensors to measure ventricular ejection and peripheral arterial pulse arrival time, respectively (20).

#### Wearables

Wearable technology is a blanket term for electronics that can be worn on the body, either as an accessory or as part of material used in clothing. One of the major features of wearable technology is its ability to connect to the internet through a smartphone, computer or tablet, enabling data to be exchanged between a network and the device. There are many types of wearable technology, but some of the most popular devices are activity trackers and smartwatches. Smartwatches are becoming increasingly popular, and 15% of the adult US population is expected to own a smartwatch in 2019 (21).

Several smartwatches that measure BP have recently been introduced. Most of these wearables measure BP through PTT from a pulse wave measurement at the wrist. The Heartisans Blood Pressure Watch (Heartisans, Hong Kong, Hong Kong) is such a smartwatch that utilizes electrocardiography and photoplethysmography to measure PTT and estimate BP. The Heartisans watch requires a motionless 20-s scan with the device held at heart level for measurements and provides systolic and diastolic BP readings. Calibration with a validated cuff-type BP device is required prior to standalone use. Measurements are synchronized to a phone application developed by the manufacturer through Bluetooth. Despite its availability on the market, the Heartisans Watch has not undergone a formal validation study.

**Figure 2:**
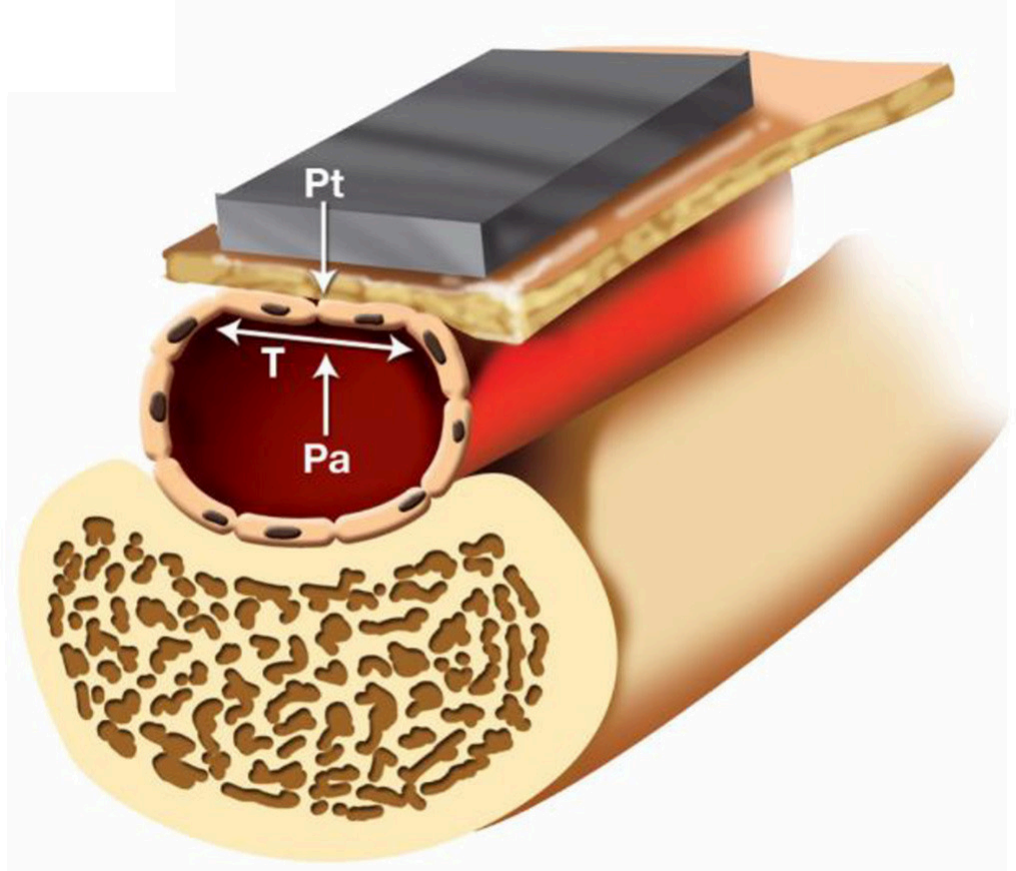
Arterial tonometry based cuff-less blood pressure measurement. First, a non-invasive arterial tonometer is placed on the skin overlying a peripheral artery. Then, the sensor applies force to compress the artery against the underlying bone until the maximum pulse pressure is achieved. At that point, it can be assumed that the only force acting on the artery is the inward force of the tonometry sensor (P_t_), the outward force of the intravascular blood pressure (P_a_), and the wall tension of the underlying artery (T). The maximum pulse pressure occurs when the artery is compressed such that the wall tension force is directed perpendicularly to the tonometry sensor and blood pressure forces. Directing the wall tension perpendicularly ensures that the tonometric force measured by the sensor only represents the blood pressure of the underlying vessel, and any deviation from this axis may introduce tangential forces and measurement error.

Another method to obtain BP measurements from the wrist with a smartwatch is through arterial tonometry (Figure 2). Arterial tonometry utilizes the concept of arterial wall applanation to measure the arterial BP waveform. The BPro device (HealthSTATS Technologies, London, United Kingdom) is a smartwatch based on tonometry. The tonometer's force transducer is integrated into the wrist strap of the watch and compresses the tissue and radial artery enough to cause applanation, while a monitor displays readings to the patient and wirelessly transmits data to a secure web portal that can be accessed by the clinician. The BPro is marketed to Family and General Practitioners as an alternative to conventional ambulatory cuff BP monitors and comparatively allows for continuous 24-h waveform analysis of BP vs. traditional 20-min interval snapshot measurements. Calibration to the brachial artery BP is required using a validated upper arm cuff device prior to use. To avoid a loss of coupling to the radial artery during measurement, the BPro requires fixation with adhesive tape prior to the 24-h measurement period by the prescribing physician. The BPro has been validated in a non-ambulatory study following the Association for the Advancement of Medical Instrumentation (AAMI) standards and the European Society of Hypertension (ESH) protocol (22). The AAMI standard requires a mean BP difference of ≤5 mmHg with a standard deviation of ≤8 mmHg vs. auscultatory reference measurement, whereas the ESH protocol requires that the majority of subjects have investigational BP readings within ≤5 mmHg of the reference measurement. While the BPro device met these requirements in a static environment, the technology is adversely affected by motion artifact and demonstrated only modest agreement with a standard automated oscillometric cuff in the ambulatory setting (23).

#### Medical “Tricorders”

The idea of a medical tricorder comes from an imaginary device on the science fiction TV show Star Trek from the 1960s which featured fictional character Dr. Leonard McCoy using it to instantly diagnose medical conditions (24). In the present time, a medical tricorder is a handheld portable device to be used by consumers to self-diagnose medical conditions and take basic vital signs measurements. Compared to wearable devices and mobile phones, medical tricorders are not “everyday” items already used (i.e., smartphone, watch); however, their relatively small size and user-friendliness still make them advantageous relative to cuff based monitors.

Several manufacturers have recently introduced medical tricorders that measure BP among other vital signs. The BodiMetrics Performance Monitor (BodiMetrics LLC, Manhattan Beach, California, United States) uses electrocardiogram and photoplethysmograph data to estimate BP through PTT. The Bodimetrics tricorder obtains measurements with a 20-s scan of the user's fingertip at heart level but only provides systolic BP data. Calibration to a validated cuff-type device is required prior to standalone use. While the Bodimetrics Performance Monitor has not been formally validated, it has been compared to an automated sphygmomanometer in an unconventional study (25). The observed large spread in absolute bias vs. an automated sphygmomanometer seems to indicate that the BodiMetrics Performance Monitor is unlikely to meet accuracy and precision standards in a formal validation study. The Bodimetrics tricorder data can be transferred to a smartphone app through Bluetooth.

Another commercially available tricorder is the FreeScan Personal Cardiovascular Monitor (Maisense Inc., Zhubei City, Taiwan). This device estimates BP from PTT as well; however, the device is unique in that it utilizes a force sensor to capture the systolic arterial waveform rather than photplethysmography. This requires the user to physically apply the force sensor directly to the radial artery for around 10 s for measurements. The Freescan device has undergone a formal validation according to the AAMI standards and met the validation criteria (26). The Freescan tricorder data can be transferred to a smartphone app and a cloud-based database through Bluetooth and the internet. Physicians also can sign up to receive warnings from the cloud-based database when abnormal BP values are detected.

Lastly, the SOMNOtouch NIBP is a non-traditional medical tricorder that utilizes PTT data collected in a similar fashion to the Bodimetrics Performance Monitor. However, it is “non-traditional” in the sense that it is worn on the wrist, and photoplethysmography data is collected from a finger clip. This setup is advantageous because it allows for beat-to-beat continuous BP measurement, similar to the BPro device, which may have independent prognostic significance (27). Built-in Bluetooth allows for easy transfer of data to a PC or mobile phone. The SOMNOtouch NIBP has been validated according to the ESH protocol; however, it demonstrated poor agreement at higher SBP and DBP values (28).

### Current Drawbacks of Cuff-Less BPT

Several limitations pertain to the currently available cuff-less BP technology.

First, the majority of smartphone applications, wearables, and tricorders have not undergone a validation protocol, and as such, their accuracy and precision is unconfirmed. The devices that have been validated using the static AAMI or ESH protocol may not have the same accuracy in the ambulatory setting (23, 28). Importantly, the AAMI and ESH validation protocols were developed to validate non-invasive cuff-type BP monitors and are based on an underlying assumption that the tested device is capable of measuring BP without an initial calibration reference measurement. Since the calibration measurement for cuff-less monitors is typically completed just prior to the conventional, static validation protocol, it is unlikely that significant variation in BP from this calibration measurement occurs during a validation series within a subject (23, 25, 26). In fact, significant variation in BP measurements (>12 mmHg systolic or >8 mmHg diastolic) from the validated reference device is an exclusion criterion in the AAMI protocol. In real life, a cuff-less BP monitor should operate accurately over a range of pressure after being calibrated at a certain pressure, but this is not tested when using current protocols. It is paramount that cuff-less devices are accurate- as inaccurate measurements may lead to false patient reassurance, unnecessary health care utilization, or misguided self-titration of hypertension medications. Misdiagnosis of hypertension could also lead to psychological stress and unneeded exposure to adverse side effects of medication. To highlight the potential scope of such problems, an error range of just ±5 mmHg could lead to an estimated 21 million normotensive and 27 million hypertensive patients being misclassified (29).

Second, the current measurement methods based on PTT and tonometry are sensitive to motion artifact and loss of signal upon movement. In several of the aforementioned studies on cuff-less PTT-based devices, researchers encountered a high rate of measurement errors and calibration errors (25, 26). Tonometry-based cuff-less devices need to be tightly coupled to the underlying artery usually with an adhesive tape to prevent loss of coupling and loss of measurements (23).

Additionally, the software and data transfer on the back end of most of the cuff-less BP measurement modalities is critically underdeveloped. This is a clinically relevant concern, since frequent BP self-measurement without a co-intervention from a healthcare provider does not seem to affect BP control much (10).

Finally, for all of the current cuff-less BPT technologies, it is presently unclear how the measured values in real life will relate to clinical or cuff-based home BP measurements. Because cuff-less methods make it much easier to perform measurements “on the go,” it is likely that these measurements are substantially different from resting BP values. Prospective clinical studies would need to assess how these measurements compare to conventional BP measurements, and what values or trends are ultimately associated with adverse outcomes. This would provide a scientific basis for medication adjustments using cuff-less BPT.

## Future Directions

To overcome the issues related to extending the use of validation protocols developed for cuff-based devices to the validation of cuff-less BP monitors, the Institute of Electrical, and Electronics Engineers (IEEE) has proposed a validation protocol specifically for cuff-less BP monitors (30). This validation protocol requires the same accuracy and precision for cuff-less monitors as the AAMI standard for cuff-based devices. The IEEE protocol differs from the AAMI/ESH protocols in that it includes validation measurements after artificial changes in BP within a subject after initial calibration to ensure accuracy over a wide range of BP values. Additionally, the IEEE protocol includes validation measurements obtained after a significant time period (weeks to months) since the initial calibration to investigate time-dependent calibration integrity.

PTT-based BP measurement is currently an active area of research, and many groups have developed methods that may help improve the accuracy of future PTT-based cuff-less BP measurement devices. For example, BP estimations can be improved by measuring PTT through plethysmography between two distal points, rather than from an electrocardiogram and one distal point (31). To minimize the influence of cardiac pre-ejection period differences, ballistocardiography (heart sound) sensors may be useful (32). To reduce the confounding effect of smooth muscle contraction and relaxation in small arteries on PTT, there have been attempts to measure PTT in more central arteries, for example, by using radar (33). Interestingly a recent study developed a smartphone case that appears to be able to measure BP accurately using a method combining photoplethysmography and tonometry (34).

If accurate and user friendly cuff-less smartphone applications, wearables, or tricorders can be developed in the future, there are several subsequent steps that would need to be taken before they can be implemented in clinical BPT practice. First, safe, secure, and efficient Cloud-based data storage would need to be developed that is easy to access for healthcare providers. Then, appropriate prospective clinical studies would need to assess what values are actually abnormal when using cuff-less measurements and what values are ultimately associated with adverse outcomes. If BPT with cuff-less measurement eventually becomes practical for managing hypertension and preventing cardiovascular events, development of appropriate reimbursement structures is needed to allow physicians to implement its use.

## Conclusions

The availability of simple, accurate, and affordable cuff-less BP devices has the potential to greatly increase the utilization of BPT in the management of hypertension. As the field of connected healthcare continues to expand, further research is needed to improve the current smartphone, wearable, and tricorder cuff-less BP technologies. Despite promising developments and preliminary data, many of the current cuff-less BP devices lack proper validation and have limited practical use due to motion artifact, a lack of proper back-end databases, and a lack of reimbursement models. Future validation studies should utilize a cuff-less BP monitor specific protocol which addresses the potential issues of calibration procedures and the stability of measurements over time. If accurate and user friendly cuff-less monitors can be developed, clinical studies are required to establish the clinical significance of this type of measurement for BPT in concurrence with the development of appropriate online databases and proper reimbursement structures.

## Author Contributions

DB and NvH wrote the body of the manuscript. All authors equally contributed to revisions and edits to the article. All authors conceptualized the article.

### Conflict of Interest Statement

JJ is a founder, equity owner, and has received research support from RTM Vital Signs, LLC, a company developing non-invasive and long-term implantable vital sign monitoring systems. RTM Vital Signs is not associated with any devices discussed in this manuscript. The remaining authors declare that the research was conducted in the absence of any commercial or financial relationships that could be construed as a potential conflict of interest.
